# Germline variant in *MSX1* identified in a Dutch family with clustering of Barrett’s esophagus and esophageal adenocarcinoma

**DOI:** 10.1007/s10689-017-0054-2

**Published:** 2017-11-13

**Authors:** A. M. J. van Nistelrooij, R. van Marion, W. F. J. van Ijcken, A. de Klein, A. Wagner, K. Biermann, M. C. W. Spaander, J. J. B. van Lanschot, W. N. M. Dinjens, B. P. L. Wijnhoven

**Affiliations:** 1 Department of Surgery, Erasmus MC Cancer Institute, University Medical Center Rotterdam, P.O. Box 2040, 3000 CA Rotterdam, The Netherlands; 2 Department of Pathology, Erasmus MC Cancer Institute, University Medical Center Rotterdam, Rotterdam, The Netherlands; 3 Center for Biomics, Erasmus MC Cancer Institute, University Medical Center Rotterdam, Rotterdam, The Netherlands; 4 Department of Clinical Genetics, Erasmus MC Cancer Institute, University Medical Center Rotterdam, Rotterdam, The Netherlands; 5 Department of Gastroenterology and Hepatology, Erasmus MC Cancer Institute, University Medical Center Rotterdam, Rotterdam, The Netherlands

**Keywords:** Barrett’s esophagus, Esophageal adenocarcinoma, Familial clustering, Exome sequencing, Genetic testing

## Abstract

The vast majority of esophageal adenocarcinoma cases are sporadic and caused by somatic mutations. However, over the last decades several families have been identified with clustering of Barrett’s esophagus and esophageal adenocarcinoma. This observation suggests that one or more hereditary factors may play a role in the initiation of Barrett’s esophagus and esophageal adenocarcinoma in these families. A Dutch family with clustering of Barrett’s esophagus and esophageal adenocarcinoma was identified. Normal DNA obtained from the proband diagnosed with Barrett’s esophagus was analyzed with SNP array and exome sequencing. A custom-made panel consisting of potential germline variants was verified in the normal DNA of the affected family members. In addition, the respective tumors were analyzed for somatic loss of the wild type allele or the presence of an inactivating somatic mutation in the wild type allele. Exome sequencing revealed 244 candidate variants in the normal DNA of the proband, of which 212 variants were verified successfully. After the normal DNA of the affected family members was analyzed for the presence of the 212 potential germline variants and subsequently the respective tumors, only one potential germline variant in *MSX1* (chr4: 4861985 T > G, c.359T > G, p.V120G, NM_002448) showed loss of the wild type allele in the tumor DNAs of the affected family members. A germline variant in *MSX1* was identified in a Dutch family with clustering of Barrett’s esophagus and esophageal adenocarcinoma. This finding indicates that the germline defect in *MSX1* may be associated with Barrett’s esophagus and cancer in this particular family.

## Introduction

Esophageal adenocarcinoma (EAC) is a histopathological subtype of esophageal cancer, of which the incidence is rising rapidly over the last decades in Western countries [1–3]. The presence of Barrett’s esophagus (BE), as a consequence of chronic gastro-esophageal reflux (GER), is generally accepted as the predominant risk factor of EAC. Other risk factors are high age, male gender, Caucasian ethnicity [4], and obesity [5]. The risk of developing EAC from BE is estimated at 0.12–0.5% per year [6, 7] and follows a multimorphological sequence, in which metaplasia evolves to low-grade dysplasia (LGD), high-grade dysplasia (HGD) and ultimately into invasive adenocarcinoma [8].

Although the vast majority of BE and EAC cases are sporadic and caused by somatic mutations, over the last decades several families have been identified with clustering of BE and EAC [9–12]. Previous studies introduced the definition familial BE (FBE), i.e. two or more first- or second-degree family members diagnosed with BE or EAC. It has been estimated that approximately 7% of BE and EAC cases are considered familial [13–15]. When compared to sporadic cases, familial cases of BE and EAC are mostly diagnosed at a younger age [14–16]. It can be hypothesized that the initiation of BE and EAC in several members of one family is caused by the presence of one or more inherited factors. The pattern of inheritance of most familial cancer syndromes is based on the concept of “Knudsons two hit” hypothesis, causing a phenotypic dominant inheritance pattern, i.e. biallelic inactivation of a tumor suppressor gene caused by one germline mutation inherited from one parent followed by a somatic second inactivating mutation in the wild type allele. Based on the pattern of inheritance suggested for FBE [17], it is likely that development of familial BE is caused by an inherited germline mutation in a (unknown) tumor suppressor gene followed by a somatic second inactivating mutation in the wild type allele causing biallelic inactivation.

Orloff et al. identified germline mutations in the genes *MSR1, ASCC1*, and *CTHRC1* with the use of a linkage analysis on affected siblings diagnosed with BE or EAC [18]. However, information about the presence of identical germline mutations in affected siblings is lacking and the role of these genes in the development of FBE is unknown. Extensive candidate gene and linkage researches have to date been unsuccessful in identifying genetic variants that are associated with the risk of FBE.

Here, we describe a family, of whom two members were diagnosed with BE and three with EAC. To identify a possible germline defect in the affected family members, we investigated the normal DNA of a proband with a SNP array and exome sequencing. Subsequently, we validated the potential germline variants identified in the proband in the normal and tumor DNA of the other affected family members on a Next-Generation sequencing platform.

## Methods

This study was approved by the Erasmus MC—University Medical Center Rotterdam Institutional Review Board. Formal written informed consent was obtained from the living family members, whom are therefore included in the study. All tissues investigated in this study were used in accordance with the code for adequate secondary use of tissue, code of conduct: “Proper Secondary Use of Human Tissue” as established by the Dutch Federation of Medical Scientific Societies (http://www.federa.org).

### Family presentation

**Fig. 1:**
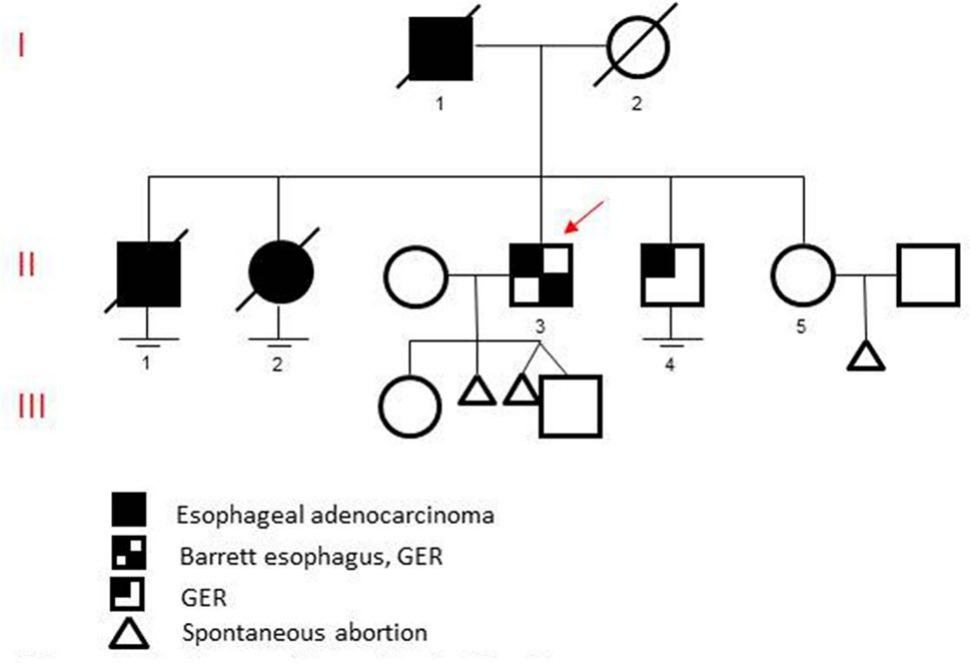
Pedigree of investigated family. Black symbols indicate esophageal adenocarcinoma. Partly blocked symbols indicate Barrett esophagus and/or gastro-esophageal reflux (GER). The proband is indicated by a red arrow

The first family member (further referred to as proband), who came to our attention is a male patient of 45 years (Fig. 1: II.3). He has been suffering from pyrosis for several years and was diagnosed in … with BE (LGD) based on histopathological examination of multiple biopsies obtained during upper gastrointestinal endoscopy. In addition, in his family there is a high incidence of BE and EAC (Fig. 1). His father (I.1) was diagnosed, in 1982 at the age of 50 years, with EAC based on histopathological examination of multiple biopsies during upper gastrointestinal endoscopy, unfortunately he died in the same year of the consequences of the disease (no tissue available). The oldest brother of the proband (II.1) was diagnosed with EAC at the age of 49 years. He underwent neoadjuvant chemoradiotherapy followed by an esophagectomy. Pathological examination of the resection specimen revealed a vital adenocarcinoma within the background of BE (HGD) located at the distal esophagus, Mandard score III–IV, ypT3N0. He died 1 month later at the age of 50 years due to post-operative complications. At autopsy, liver metastases were identified. Furthermore, the older sister of the proband (II.2) was diagnosed with a poorly differentiated adenocarcinoma at the gastro-esophageal junction at the age of 45 years and died 1 year later of the consequences of the disease. The younger brother of the proband (II.4) has been suffering from pyrosis for several years, and was diagnosed with BE (intestinal metaplasia, no dysplasia) based on histopathological examination of multiple biopsies obtained during upper gastrointestinal endoscopy at the age of 40. He was prescribed proton pump inhibitors and included in the surveillance program for BE, in the biopsies taken during the latest control no intestinal metaplasia was observed any longer. The youngest sister of the proband (II.5) did not give informed consent, and was therefore not included in the study.

### SNP array and exome sequencing

The proband (II.3) was subjected to genetic testing, i.e. SNP array and exome sequencing performed at the Erasmus MC Center for Biomics, Rotterdam, the Netherlands. After informed consent was obtained during a counselling session at the Department of Clinical Genetics, blood was drawn and DNA was isolated using a Chemagic DNA Blood Kit according to standard procedures. To identify copy number variations (CNVs) in the germline DNA of the proband (II.3), the Genome-wide human SNP array 6.0 was performed according to the manufacturer’s protocol (Affymetrix, Santa Clara, CA, USA). Data were analyzed using Nexus Copy Number tm V4 software (Biodiscovery, Hawthorne, CA, USA). Exome sequencing was performed on the Hiseq2000, using the Agilent version 4 capture kit, according to the Illumina TruSeq v3 protocol. Reads were aligned against the human reference genome build 19 (hg19) using Burrows-Wheeler Aligner [19] and the NARWHAL pipeline [20]. Subsequently, genetic variants were called using tools from the genome analysis toolkit [21]. The resulting VCF files were processed with a custom variant annotation tool that determines the variant effects. Germline variants identified by exome sequencing were selected to cause amino-acid changes or splice-site alterations, in addition variants present in the dbSNP135 database or with a frequency of > 1% in ESP6500 and the 1000 Genomes databases were excluded. To confirm these data on a different platform, a custom-made panel was designed by the Ion AmpliSeq Designer V2.0 for targeted sequencing on the Ion Torrent Personal Genome Machine (Ion PGM) (Life Technologies, Carlsbad, CA, USA). In short, libraries were made using the Ion AmpliSeq Library Preparation Kit. A template was prepared using the Ion OneTouch Template Kit and sequencing was performed with the Ion Sequencing Kit v2.0 on an Ion 316 chip. Data were analyzed with the Variant Caller v2.2.3-31149 (Life Technologies, Carlsbad, CA, USA). For the variants that could not be validated by the ion PGM, primers were designed for PCR amplification and Sanger sequencing on an ABI 3730 sequencer (Life Technologies, Carlsbad CA, USA) according to the BigDye Terminator v3.1 Cycle Sequencing Kit Protocol. DNA sequences were visualized using the Mutation Surveyor software (Softgenetics, State College, PA, USA), which aligned the sequences to annotated GenBank reference files.

### Tissue samples

To test whether the validated germline variants in the proband (II.3) could also be identified in the normal and tumor DNA of the family members, formalin-fixed paraffin-embedded (FFPE) tissue blocks were reclaimed from several pathology archives in the Netherlands. Tumor tissue (II.1, II.2) composed of at least 50% neoplastic cells (confirmed by a GI-pathologist) and areas of non-malignant esophageal cells (II.1, II.2, II.4) were manually microdissected from 10 to 15 hematoxylin-stained sections (4 µm). Subsequently, DNA was extracted using proteinase K and 5% Chelex 100 resin.

## Results

**Fig. 2:**
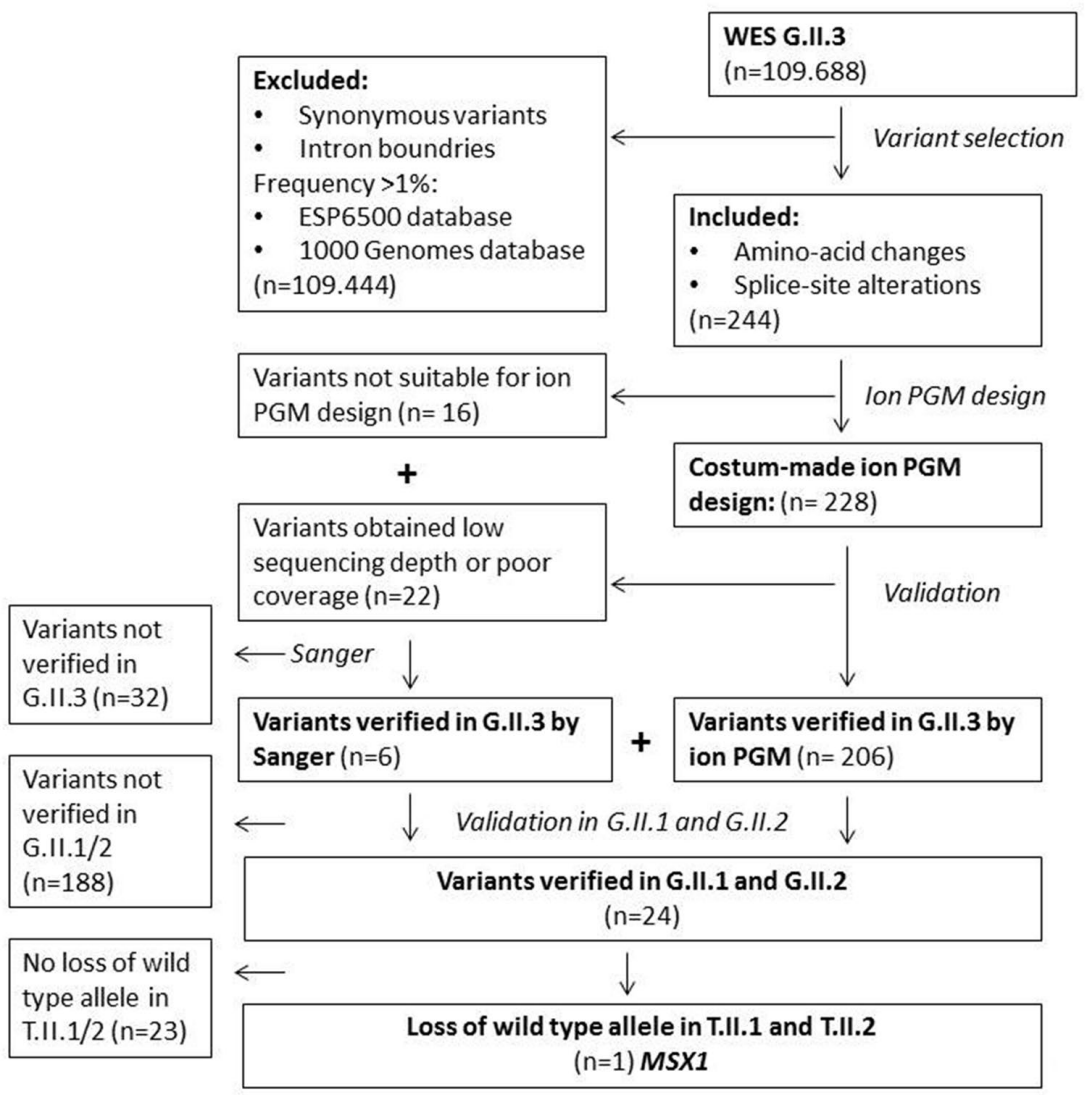
Flowchart of sequencing pipeline *WES* whole exome sequencing, *Ion PGM* Ion Torrent Personal Genome Machine, *Sanger* Sanger sequencing, *G* Germline DNA, *T* Tumor DNA

The SNP array revealed a known CNV with no clinical relevance and a CNV in an unknown gene (data not shown). Exome sequencing performed on the normal DNA of the proband (II.3) revealed after the selection procedure 244 candidate germline variants (Table 1). For 228 variants custom-made primers could be designed to confirm the data on a different platform. After sequencing on the ion PGM, 206 variants were verified successfully in the normal DNA of the proband (II.3). The remaining 22 variants obtained either low sequencing depth or poor coverage and therefore did not qualify for further analysis on the ion PGM. For the 38 variants, which could not be validated by the ion PGM, primers were designed for PCR amplification and Sanger sequencing. Of the 38 variants, six were validated in the normal DNA of the proband (II.3) by Sanger sequencing. Leaving 212 potential germline variants of interest, which were validated in the family members (Fig. 2).

**Table 1:** Mutations identified in normal DNA of the proband by exome sequencing

	Amount N = 244 (%)
Non-synonymous	164 (67.2)
Stop gains	3 (1.2)
Frameshift indels^a^	14 (5.7)
Non-frameshift indels^a^	44 (18.0)
Splice-site variants	12 (4.9)
Unknown	7 (2.9)

^a^ *Indels* insertions and deletions

**Fig. 3:**
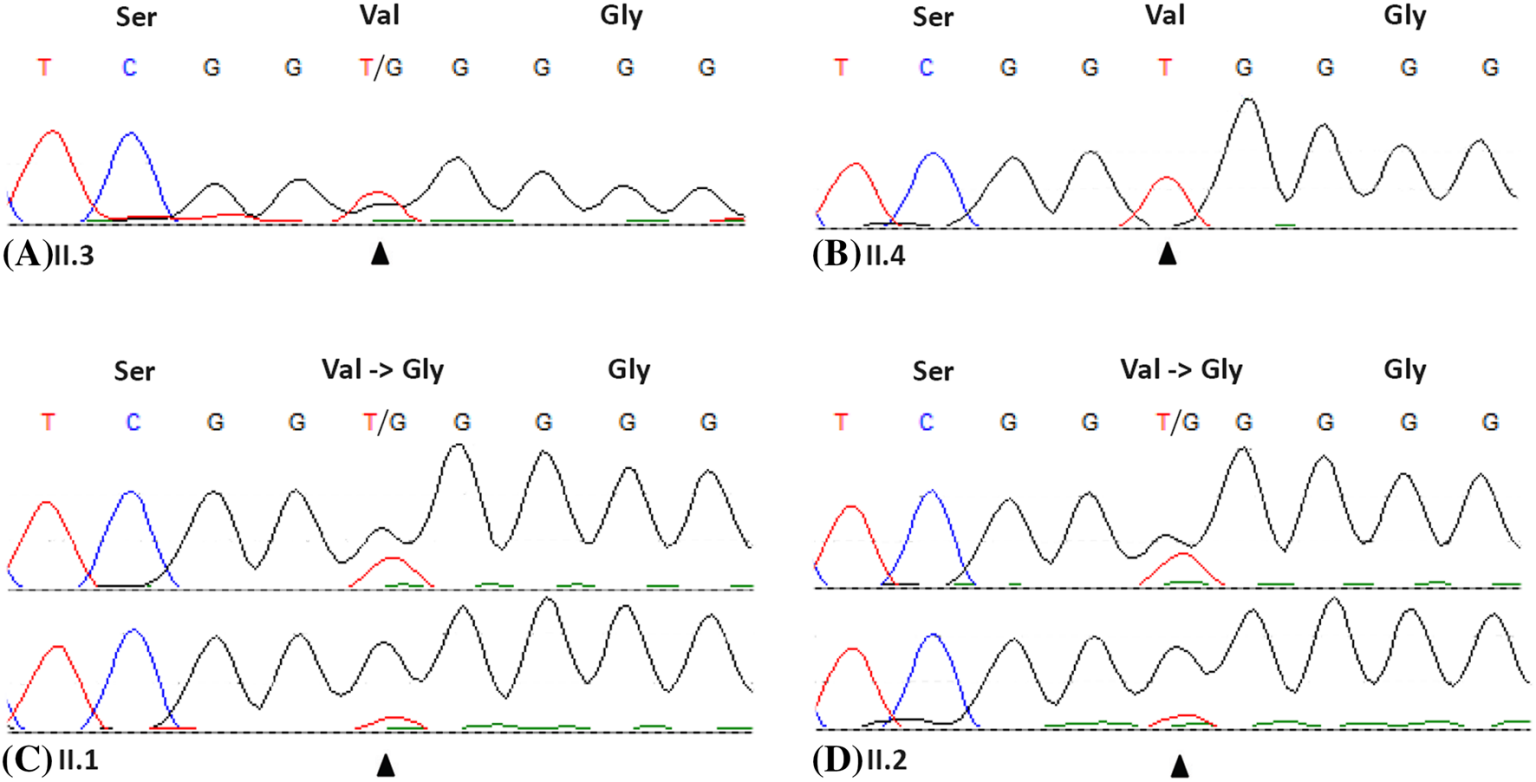
Sanger sequencing results of *MSX1*. **a** Sanger sequencing of normal DNA of the proband (II.3) confirmed the presence of the heterozygote variant (c.359T > G) in *MSX1*. **b** Sanger sequencing of normal DNA of youngest brother of the proband (II.4) revealed no variant in *MSX1*. **c** Sanger sequencing of normal DNA (upper panel) and tumor DNA (lower panel) of the oldest brother of the proband (II.1) confirmed the presence of the heterozygote variant (C.359T > G) in *MSX1* in normal DNA and loss of the wild type allele in tumor DNA which changed the codon 120 from Valine into Glycine (P.V120G). **d** Sanger sequencing of normal DNA (upper panel) and tumor DNA (lower panel) of the older sister of the proband (II.2) confirmed the presence of the heterozygote variant (c.359T > G) in *MSX1* in normal DNA and loss of the wild type allele in tumor DNA, which changed the codon 120 from Valine into Glycine (p.V120G)

The custom-made panel for the ion PGM, containing the germline variants identified in the proband (II.3), was extended to two other affected members of the family (II.1, II.2). Normal DNA of the affected family members (II.1, II.2) was analyzed for the presence of the 212 potential germline variants identified in the proband (II.3). In addition, the respective tumors of the family members (II.1, II.2) were analyzed for somatic loss of the wild type allele or the presence of an inactivating somatic mutation in the wild type allele. Twenty-four of the potential germline variants were also identified in the normal DNA of the family members II.1 and II.2. Only one potential germline variant, *MSX1* (chr4: 4,861,985 T > G, c.359T > G, p.V120G, NM_002448), showed loss of the wild type allele in both the tumor DNA of family members II.1 and II.2. In the normal and metaplastic DNA of the youngest brother (II.4) no variant in *MSX1* was identified (Fig. 3).

## Discussion

For the first time a germline variant in the *MSX1* gene was identified in a family with clustering of BE and EAC with the aid of exome sequencing. In addition, the investigated tumors of two affected family members showed somatic loss of the wild type allele. The germline variant in *MSX1* changed codon 120 from Valine into Glycine. Although not reported in the Cosmic database and in dbSNP135, the variant was described before on a very low frequency (allele frequency: 0.00052) [22], suggesting that this is a rare variant. This finding indicates that the germline defect in *MSX1* may be associated with the high occurrence of BE and EAC in this Dutch family.

*MSX1* encodes a homeobox protein and is involved in multiple epithelial-mesenchymal interactions. In addition, *MSX* homeobox genes are able to interact with bone morphogenetic proteins (BMPs), in particular to the closely related BMP-2 and BMP-4. Recent studies established e.g. BMP-4 signaling as important interconnected regulatory pathways that contribute to the early stage of the transformation of the epithelial cells of the distal esophagus from the normal stratified squamous mucosa to an intestinal columnar cell type. BMP-4 was found to be present in inflamed squamous epithelium but not in normal squamous mucosa [23]. *MSX1* may be involved in the malignant progression of BE into EAC for familial cases as well as for sporadic cases, however somatic mutations in *MSX1* were identified in a very low frequency by Dulak et al. (p.R158W, p.F151L, p.P153P) [24], suggesting a prominent role of this germline defect in the development of BE and EAC in this particular family. In addition, mutations in *MSX1* have also been reported in families with dominantly inherited congenital absence of several permanent teeth, called oligodontia or hypodontia, with or without cleft lip and/or palate [25–28], no oligodontia or hypodontia was observed in the proband.

In one of the family members (II.4) the *MSX1* variant was not observed, although he did suffer from pyrosis and was diagnosed with intestinal metaplasia at the first histopathological examination. However, the most recent biopsies taken during upper gastro-intestinal endoscopy revealed no signs of intestinal metaplasia. It can therefore be hypothesized that family member II.4 is a phenocopy. Since the prevalence of BE in the common population is estimated at 2% [29, 30], it can be anticipated that the initial metaplasia in this family member developed as a consequence of an environmental factor, instead of within the context of an inherited genetic defect.

Family member II.3 was indicated as the proband and since he was only diagnosed with BE without invasive carcinoma, it has to be taken into account that *MSX1* may only be associated with the development of BE and not necessarily with EAC. In addition, it was not possible to test the presence of the *MSX1* variant in the father of proband (I.1), also diagnosed with EAC, since no tissues blocks were present.

Previous studies introduced and persevered the definition familial BE, i.e. two or more first- or second-degree family members diagnosed with BE, and/or EAC. These studies considered familial BE and familial EAC to be part of the same genetic trait, because EAC appears to arise from BE and both conditions share the same epidemiologic risk factors. However, one can hypothesize that familial EAC can be distinct from most familial BE. Since BE is much more prevalent among the common population, familial BE does not necessarily have to be the underlying condition of familial EAC. Therefore, familial EAC might be the result of accelerated malignant progression from familial BE, or familial EAC might arise without familial BE as the premalignant condition. In both scenarios involvement of specific germline mutations driving familial EAC can be envisaged. Although, the criteria of FBE can be discussed, since this definition was frequently used in the literature it was persevered in this study to conduct uniformity.

In conclusion, a germline variant in *MSX1* was identified in the normal DNA of three affected members of a family with clustering of BE and EAC, in addition the investigated tumors showed somatic loss of the wild type allele, consistent with biallelic inactivation of a tumor suppressor gene. This germline defect may be associated with the development of BE and EAC in this family. However, functional studies have to be performed to prove any effect of this germline defect.
